# Distance-Based and Low Energy Adaptive Clustering Protocol for Wireless Sensor Networks

**DOI:** 10.1371/journal.pone.0161340

**Published:** 2016-09-22

**Authors:** Misbah Liaqat, Abdullah Gani, Mohammad Hossein Anisi, Siti Hafizah Ab Hamid, Adnan Akhunzada, Muhammad Khurram Khan, Rana Liaqat Ali

**Affiliations:** 1 Faculty of Computer Science and Information Technology, University of Malaya, Kuala Lumpur, Malaysia; 2 COMSATS Institute of Information Technology, Islamabad, Pakistan; 3 Center of Excellence in Information Assurance (CoEIA), King Saud University, Riyadh, Saudi Arabia; Nanyang Technological University, SINGAPORE

## Abstract

A wireless sensor network (WSN) comprises small sensor nodes with limited energy capabilities. The power constraints of WSNs necessitate efficient energy utilization to extend the overall network lifetime of these networks. We propose a distance-based and low-energy adaptive clustering (DISCPLN) protocol to streamline the green issue of efficient energy utilization in WSNs. We also enhance our proposed protocol into the multi-hop-DISCPLN protocol to increase the lifetime of the network in terms of high throughput with minimum delay time and packet loss. We also propose the mobile-DISCPLN protocol to maintain the stability of the network. The modelling and comparison of these protocols with their corresponding benchmarks exhibit promising results.

## Introduction

Wireless sensor networks (WSNs) have received considerable research attention along with the advancement and development of technologies in recent years [1]. WSNs are used for the low-cost and long-term monitoring of physical and dynamically changing environments [2]. Sensor networks mostly comprise mobile and stationary sensors that are randomly deployed inside the network to collect data from the surroundings via wireless communication [3] [4]. The collected data are further transmitted to one or more sinks/base stations (BS) using a single-hop or multi-hop communication protocol [5]. The rapidly generated massive volume of structured and unstructured data is known as big data, which demand a reliable and flexible storage infrastructure [6]. In WSNs, small-sized sensor nodes (SNs) are deployed with low-capacity batteries, which are irreplaceable and non-rechargeable in most cases [7]. SNs also obtain useful information from a defined network and transfer such information to the BS either directly or via a chain of cluster heads (CHs) [8].

This study investigates the efficient utilization of energy among SNs to extend the network stability period [9]. The network becomes unstable when the first SN dies [10]. SNs can work over several weeks or months to a few years without refueling given their energy-constrained nature. The transmitter and receiver consume the most amount of energy among all the functional components of an SN [11] [12]. However, SNs have limited processing, storage constraints, and short battery life span. Energy consumption and storage limitation are precarious issues for resource-constrained nonstationary WSNs [13], and a high-performance, energy-efficient data storage system can guarantee an extended battery life. Despite the recent advancements in increasing the storage capacity of a sensor device, high-current WSN applications still demand an efficient energy utilization network [14] [15].

Energy efficiency is one of the key aspects and basic concepts that should be integrated into the overall network infrastructure of multiple networking domains to comply with the changing shape of the Internet [16] [17]. However, WSN nodes operate only for a limited time because of battery constraints. Therefore, these nodes have a finite lifetime, and regularly replacing their batteries entails additional costs and complexities [18]. Although the use of large batteries can prolong the operation of WSN nodes, the burdens of size, cost, and weight will increase significantly. Alternatively, the computational power of nodes may be reduced to achieve longevity, but at the cost of computation and transmission ranges [19]. Many studies have examined methods to optimize energy efficiency to extend the lifetime of battery-powered SNs [20, 21]. These studies cover energy-aware medium access control and power-aware storage protocols [22–25]. However, the lifetime of SNs remains finite and bounded. Although these techniques improve both the application lifetime and battery replacement time of WSNs, these networks still lack sufficient energy. Other design considerations for WSNs, such as scalability [26], fault tolerance [27], operating environment, production costs [28], sensor network topology, hardware plus other constraints [29–32], and transmission media [11], can be used to compare different technologies or WSN protocols. Despite the significant role of sensors in numerous aspects of our daily life, resource poverty is the main deficiency of SNs that impedes the experiences and demands of end users.

**Fig 1:**
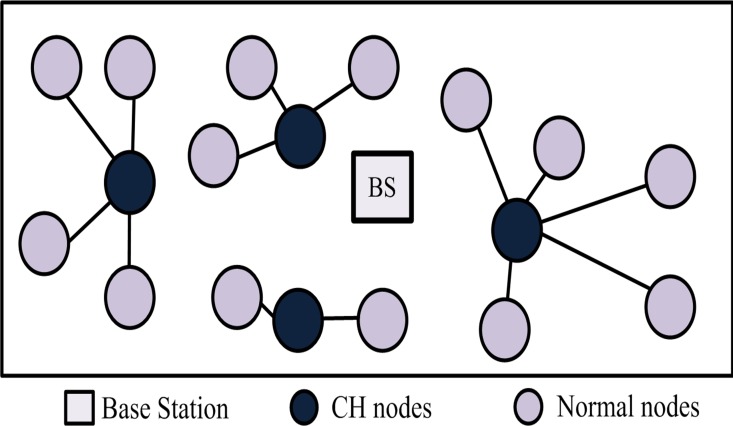
Node deployment in the conventional protocol.

Another issue in energy consumption arises from the random deployment of SNs. SNs should be deployed to cover the interested area of the entire network. Accordingly, sensors should be deployed to precise locations to ensure the accuracy of aggregated information [5]. If they remain uncontrolled, then these issues may result in unstable energy consumption and ultimately shorten network lifetime. Previous studies have focused on mitigating existing problems, and efficient power control schemes have been proposed to overcome energy issues. A review of the literature indicates that randomly deploying nodes [33–35] cannot cover the area of interest of the entire network. Furthermore, the two nearest nodes can become CHs, whose distance from each other is shorter than that between a CH and its member nodes. Moreover, non-CH nodes dissipate maximum energy when transmitting their data to CHs, thereby shortening network stability period. In existing protocols, CHs with the largest number of member nodes consume a higher amount of energy than CHs with a smaller number of SNs (Fig 1). By contrast, an improved network lifetime and a maximum throughput can be achieved if the network coverage hole is avoided by evenly distributing the SNs within the network instead of being randomly deployed.

We propose three novel energy-efficient protocols, namely, distance-based low-energy adaptive clustering (DISCPLN), multi-hop (MH)-DISCPLN, and mobile (M)-DISCPLN, to achieve efficient energy utilization in WSNs. DISCPLN handles the unequal CH selection issue and ensures high network stability and throughput based on network distribution and by adapting the concept of single-hop communication. MH-DISPCLN balances network load via hierarchical communication and extends network lifetime by minimizing the packet drop rate. M-DISCPLN collects reliable information from large-scale networks and minimizes the energy hole problem by using a mobile sink. We compare these protocols with state-of-the-art WSN protocols, such as low-energy adaptive clustering hierarchy (LEACH), distributed energy-efficient clustering (DEEC), stable election protocol (SEP), threshold-sensitive energy-efficient network (TEEN), and developed distributed energy-efficient clustering (DDEEC). The proposed protocols demonstrate promising applications in extending network lifetime and increasing throughput in both homogeneous and heterogeneous network environments.

The rest of this paper is structured as follows. The related literature is summarized in section 2. Section 3 describes the applications of the proposed protocols and the radio model. Sections 4 and 5 present our proposed protocols. Section 6 discusses the sink mobility model. Section 7 compares the overall performance of the proposed protocols with those of existing protocols. Section 8 concludes the proposed protocols.

## Related Work

The current state-of-the-art WSN technology offers a viable solution for the deficiencies in the design and development of different types of wireless sensor protocols [20]. The efficient use of energy is the most challenging issue in WSNs [36], and SNs are known for their limited battery power. The energy of a node is consumed to extend network lifetime when network data are aggregated [37]. Two types of CH schemes have been proposed based on homogeneous and heterogeneous environments to enhance network performance [25, 38]. LEACH is the first homogeneous protocol that is deployed on SNs with the same initial energy. In this protocol, data transmission undergoes two phases, namely, setup and steady-state phases [23, 39]. In the setup-phase, the nodes are randomly selected as CHs based on a certain probability, are generated using a CH selection algorithm as in LEACH [23], and are formed dynamically. In the steady-state phase, the nodes within the clusters transmit their data to the appropriate CH within a specified region, and then the CH further aggregates and transfer the received data to the BS. LEACH selects data transmission phases in each round based on their time and selects a random CH to balance the energy. However, this protocol does not guarantee the selection of an optimal number of CHs, and its performance does not improve in a heterogeneous environment.

LEACH-centralized (LEACH-C) is an extended version of LEACH in which maximum-energy nodes are selected as CHs. However, the farthest nodes with the minimum energy cannot forward their data to the BS [40]. The MH-LEACH [41] protocol is based on inter- and intra-cluster operations, but does not focus on full coverage of the network area. Alternatively, the power efficient gathering in sensor information systems (PEGASIS) protocol [24] outperforms LEACH by using chain-based transmission. In PEGASIS, data are transmitted to the BS via a chain of organized nodes. The nodes can receive and transmit aggregated data from one SN to another node, and the obtained data are received by the BS via a designated node.

In heterogeneous networks, SEP [22] has two levels of energy with normal and advanced nodes, in which each node selects itself as a CH with knowledge about the initial and current energy of the other nodes. Advanced nodes have extra energy as compare to normal nodes. Although SEP can extend network stability period, this protocol cannot do so in multilevel homogenous networks. By contrast, DEEC [25] has multilevel heterogeneous nodes that assume energy at a certain deployment time. In DEEC, CHs are randomly selected based on the node’s residual energy and the average energy of the network. Advanced nodes are selected as CHs more frequently than normal nodes. TEEN [42] is a threshold-based protocol that obtains the best network lifetime because of its reactive nature. Numerous protocols have been proposed based on LEACH, TEEN, DEEC, and SEP. For example, Q-LEACH [43] extends the lifetime of homogeneous networks. MODLEACH [44], which adopts the concept of hard and soft thresholds, can provide a longer network stability period than LEACH. In addition, [45] presented an in-depth comparison of the different variants of DEEC (i.e., DDEEC and EDEEC) in terms of their applications and energy efficiency of nodes.

LEACH [23], PEGASIS [24], DEEC [25], and SEP [22] obtain a longer stability period by considering the static network elements. However, the mobility concepts in the network are adopted to maximize network performance in terms of network throughput and stability period [46]. The sink mobility approach is proposed to stabilize energy consumption among SNs [47] [48], and a sink mobility structure is proposed using a k-level independent grid structure to transfer the data from source to the sink. Network performance is considered in [49], which uses static and mobile sink models within a predefined delay tolerance level where nodes are not required to send data as soon as they become available. By contrast, the node can temporarily store and send data when the sink is at the most suitable position to achieve reliable network performance.

## Application and First-Order Radio Model

**Fig 2:**
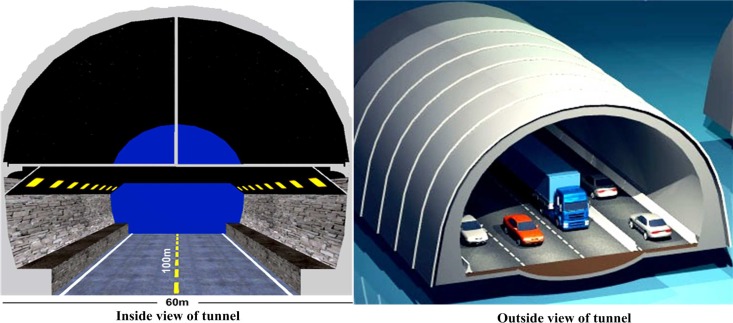
WSN tunnel-based application.

From the commercial and technological perspectives, WSNs are used in every aspect of life, such as in military monitoring sensors that are deployed to detect temperature, heat, and blood pressure [50], and in air pollution and forest fire detection sensors that are deployed to sense humidity and gases produced by fires [51] [52]. WSNs can provide novel solutions in several fields, such as in water monitoring [53], civil engineering, and healthcare [54] [55], but they primarily focus on issues related to energy consumption and require an appropriate network design. Application-oriented WSNs are designed to accomplish certain objectives. Consequently, energy-efficient routing protocols are always required to fulfil the performance requirements of the network. WSNs may also come in different shapes, such as cylindrical, rectangular, or square. We propose a rectangular network application model (Fig 2) as a tunnel for evaluating the network lifetime, throughput, packet drop, and delay time of WSNs.

### First-Order Radio Model

**Fig 3:**
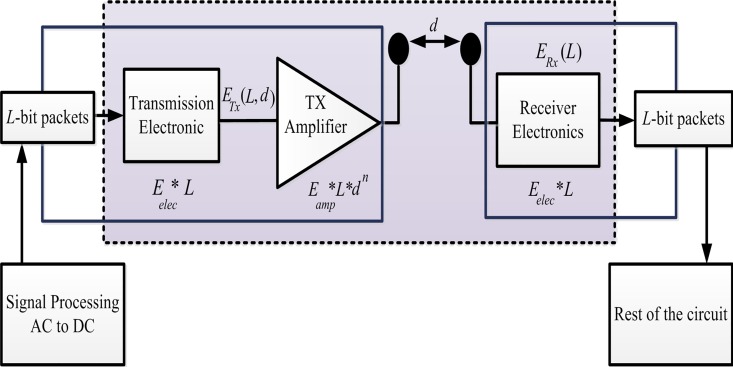
Radio energy dissipation model.

In the first-order radio model (Fig 3), energy is consumed by transmitting (L) bit messages over the distance (d) as follows [23]:

$$E_{TX}(L,d)=\{\begin{array}{ll} \;L\times E_{elec}+L\times E_{fs}\times d^{2} & if\;d<d_{0}\\ \;L\times E_{elec}+L\times E_{mp}\times d^{4} & if\;d\geq d_{0} \end{array}$$ (1)

where *E*_*elec*_ denotes the dissipated energy that is consumed to run the transmitter and receiver (*E*_*TX*_ and *E*_*RX*_, respectively). *E*_*elec*_ is based on different features, such as modulation, digital coding, filtering, and spreading of signals. *d* denotes the distance between the transmitter and the receiver, whereas *d*_0_ is calculated as *do* = *sqrt*(*E*_*fs*_/*E*_*mp*_). *E*_*mp*_ and *E*_*fs*_ depend on the distance between the transmitter and the receiver and the transmitter amplifier model. If *d* is greater than *d*_0_, then the multipath model (*d*^4^) is used. Otherwise, the free space model (*d*^2^) is used to measure the dissipated energy [56].

The performance of the proposed protocols in homogeneous and heterogeneous networks is further evaluated in the following sections.

## Proposed DISCPLN Protocol: Single-Hop Communication

**Fig 4:**
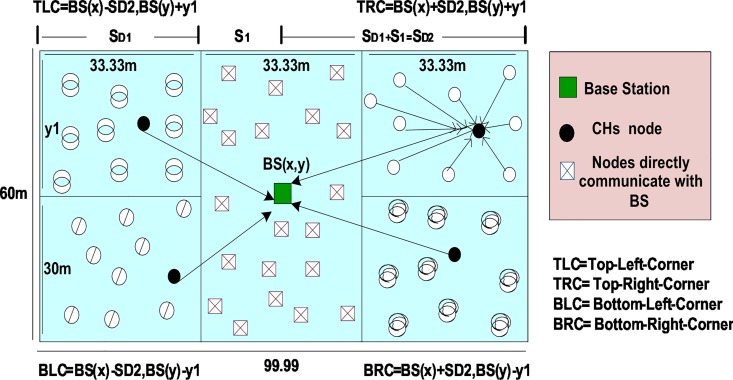
DISCPLN protocol model in homogeneous and heterogeneous environments.

An efficient protocol consumes minimum energy and achieves maximum network lifetime by covering the entire network area. A trade-off exists between network lifetime maximization and energy consumption. The network area is divided into several regions with the same number of nodes to mitigate the coverage hole problem. The distributed clustering algorithm is applied to balance the energy load in homogenous and heterogeneous WSNs (Fig 4). The DISCPLN protocol within a homogeneous environment is known as DISCPLN–LEACH, whereas those within a heterogeneous environment are known as DISCPLN–DEEC and DISCPL–P. Sections 4.2 and 4.3 discuss the CH selection schemes of these protocols.

The following section describes the function of the DISCPLN protocol, which improves network stability by distributing the entire network into five regions.

### Region Formation

In the DISCPLN protocol, the entire network area (100 m × 60 m) is divided horizontally into three equal regions. The BS is placed over the midpoint of the network, and its location is used as a reference point to define the regions in the network. Equal nodes are deployed in each sector to ensure full coverage. The nodes in the internal region directly transmit their data to BS.

The four-corner (x- and y-axes) coordinates of the network area are computed as follows:

Top-left corner (TLC) of the network boundary:

$$TLC=BS_{x}-S_{D2},BS_{y}+y_{1},$$ (2)

top-right corner (TRC) of the network boundary:

$$TRC=BS_{x}+S_{D2},BS_{y}+y_{1},$$ (3)

bottom-left corner (BLC) of the network boundary:

$$BLC=BS_{x}-S_{D2},BS_{y}-y_{1},\;\text{and}$$ (4)

bottom-right corner (BRC) of the network boundary:

$$BRC=BS_{x}+S_{D2},BS_{y}-y_{1},$$ (5)

where *BS*_*x*,*y*_ is the location of the BS, *y*_1_ is the vertical distance, and *S*_*D* 2_ is the horizontal distance from the BS to an outer boundary of the network (Fig 4). The factors *y*_1_ and *S*_*D* 2_ will be multiplied to draw more outer regions and to expand the area of the network. The following sections describe the network environment and the protocols for selecting CHs.

### Homogeneous Environment

To evaluate the performance of DISCPLN–LEACH, its simulation results are compared with those of LEACH and MODLEACH.

#### Cluster head selection

The operations of DISCPLN–LEACH are the same as those of LEACH [11]. However, in the setup phase, only one node is selected as a CH in each region based on a certain probability. Each node generates a random number between 0 and 1. If the number that is generated by a node is less than the specified threshold value *T*(*n*), then this node is selected as the CH and its message is broadcast to other nodes for membership. T(n) is calculated as follows:

$$T(n)=\{\frac{P}{1-P\times [r[mod(1/P)]]},n\in G,$$ (6)

where *P* is the total number of CHs in the area of interest, *r* denotes the number of rounds, *r*[*mod*(1/*P*)] denotes the number of nodes that are selected as CHs in a certain number of rounds, and *G* denotes the nodes that are not selected as CHs. After selecting CHs in the outer regions, the nodes directly send their data to a specific region in which nodes are deployed. As shown in the DISCPLN model, the nodes at the top-left region only transmit data to CHs within the same region instead of those within the bottom-left region (Fig 4).

### Heterogeneous Environment

In a heterogeneous environment, the network is based on advanced and normal nodes. SNs with different energy levels are deployed upon reaching depletion time. Advanced SNs are equipped with *E*_0_ (1 + *α*_*i*_) energy, where *E*_0_ is the energy of the normal node. Advanced nodes have *α*_*i*_ times higher energy than the normal node. The total energy of multilevel heterogeneous networks is computed as follows:

$$E_{total}=\sum_{i=1}^{N}E_{0}(1+\alpha_{i})=E_{0}(N+\sum_{i=1}^{N}a_{i}),$$ (7)

where *E*_*total*_ denotes the total energy of N nodes within the network.

#### Cluster head selection

**Fig 5:**
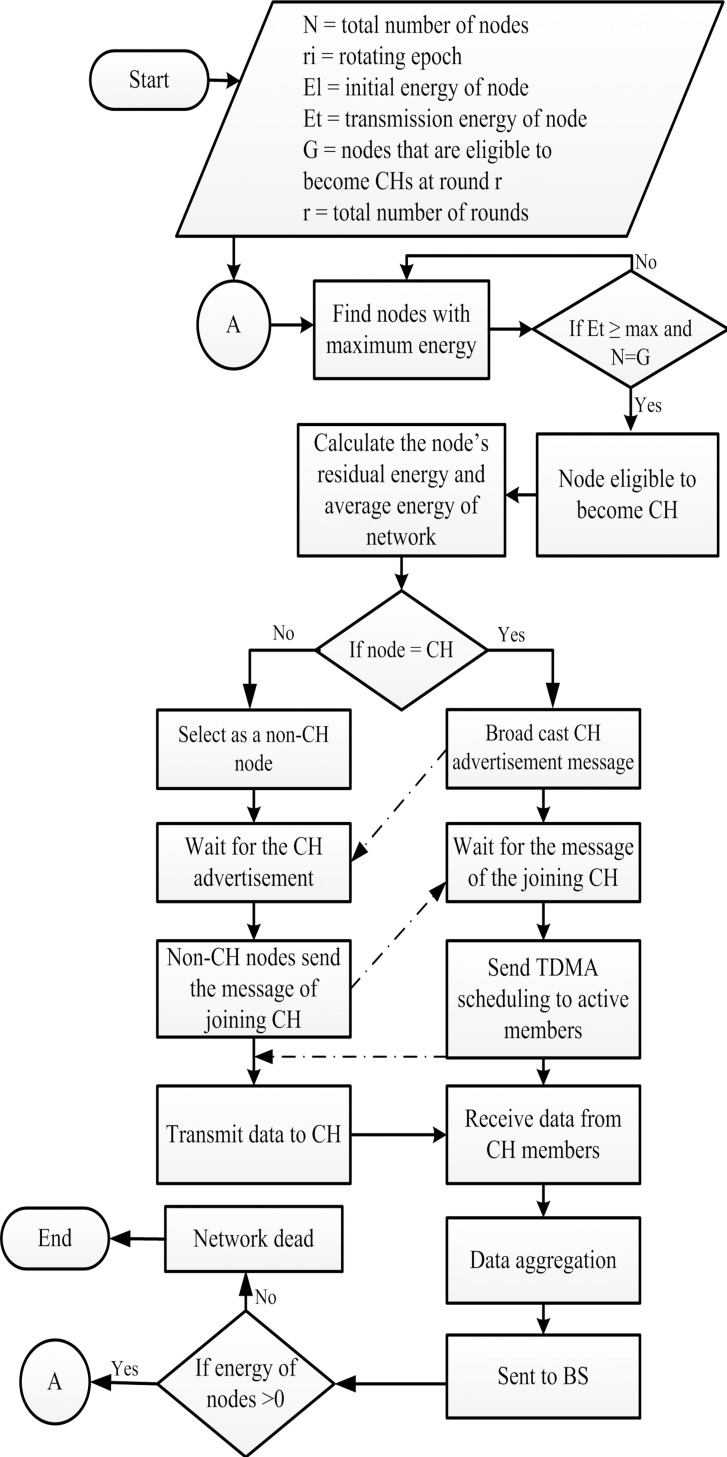
DISCPLN–DEEC CH selection.

The node will be selected as a CH in each region except for the internal region; thus, we modify DISCPLN into DISCPLN–DEEC because the fixed number of CHs in each region has a similar network deployment structure. In DISCPLN–DEEC, residual energy-based static clustering and dynamic CH selection are adopted instead of probabilistic CH selection in each round (Fig 5). DISCPLN–P is a modified version of DISCPLN that adopts the probabilistic CH approach of DEEC [25].

### Simulation Parameters and Results

Five parameters, namely, alive nodes (network stability period), dead nodes, throughput, packet delay time, and number of packet drops across a channel, are used to measure and analyse the performance of the proposed protocols. For accuracy, the simulations are executed five times, and then the average value along the confidence interval is calculated. For consistency, the same simulation parameters are used for all the protocols (Table 1).

**Table 1:** Simulation parameters.

Parameters	Values
Network Size	100 m × 60 m
Sensor Nodes	60
BS	(50 m, 30 m)
Packet Size	4000 bits
Initial Energy (*E*_0_)	0.5 J
Dissipated Energy per Bit (*E*_*elec*_)	50 nJ/bit
Probability of Packet Drop	0.3

#### Lifetime of the network

**Fig 6:**
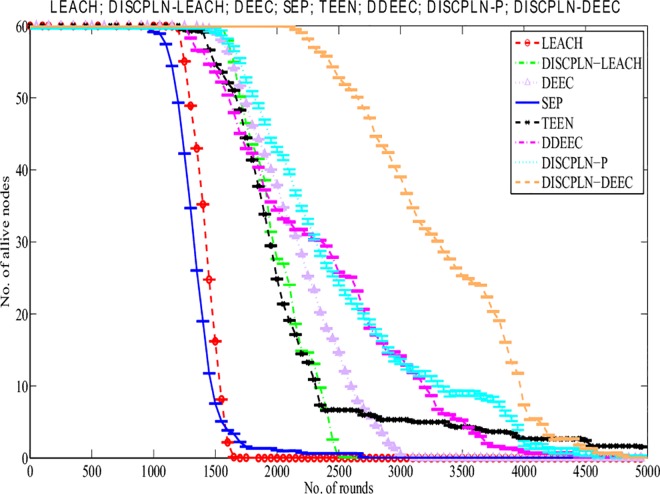
Performance comparison based on stability period for single-hop communication.

The simulation results presented in Fig 6 compare the performances of the homogeneous (i.e., LEACH and DISCPLN–LEACH) and heterogeneous protocols (i.e., DDEEC, DEEC, SEP, TEEN, DISCPLN–DEEC, and DISPLN–P) in extending the network stability period (all SNs are alive) and network lifetime (number of alive and dead nodes). For the homogenous protocols, LEACH achieves the shortest network lifetime, and DISCPLN–LEACH achieves the longest stability period. The stability period of the network refers to the entire lifetime of the first node of the network.

Data transmission energy is inversely proportional to energy depletion, and a sensor dies upon depleting all of its energy. DISCPLN–DEEC achieves the longest stability period among all the protocols because of its uniform clustering. However, given the non-uniform energy in unstable regions, the death rates of DDEEC, TEEN, DISCPLN–DEEC, and DISCPLN–P are vastly different from those of SEP and DEEC. SEP has a wide unstable region because of its threshold-based strategy. DISCPLN–P is the second best protocol in terms of the number of alive nodes during the stability period. The proposed protocols limit energy consumption because of network formation and the shorter communication distance from the internal and external region nodes to the BS. In DISCPLN–DEEC, only one node is selected as the CH in the desired region, and this node will aggregate and send its own data, along with those of its member nodes, directly to the BS.

#### Throughput of protocols

**Fig 7:**
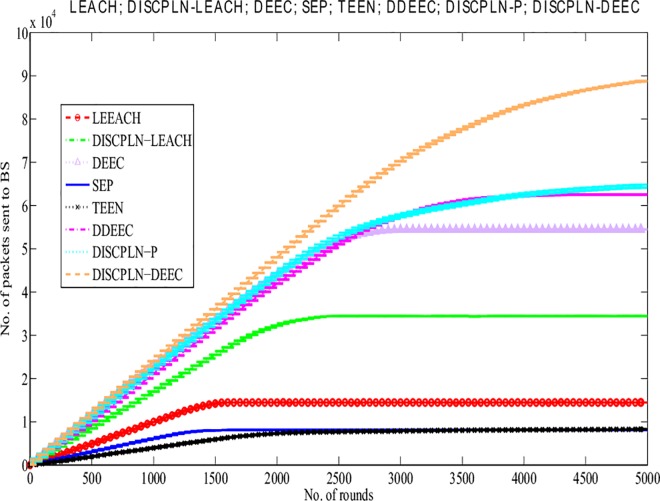
Performance comparison based on throughput for single-hop communication.

In addition to network lifetime, network throughput is another metric that is used to evaluate the efficiency of protocols. The BS confirms the efficiency of the routing protocol when receiving more data packets from CHs and nodes. The simulation results for LEACH and DISCPLN–LEACH indicates that the former protocol achieves the maximum throughput because of its network lifetime (Fig 7). This comparison result clearly supports the enhanced performance of DISCPLN–DEEC. The behavior of DISCPLN–DEEC differs from the transition state to the steady state as a result of the CH selection in each region to balance data load. Moreover, in DISCPLN–DEEC, the nodes in the central region transmit data continuously to the BS without having any CH. DISCPLN–DEEC also outperforms DDEEC, SEP, and TEEN in terms of throughput. Although the throughput of DISCPLN–DEEC is the same as that of DEEC, the throughput of the former becomes higher than that of DEEC after several iterations because of its uniform node distribution and CH selection scheme.

#### Packet drop

**Fig 8:**
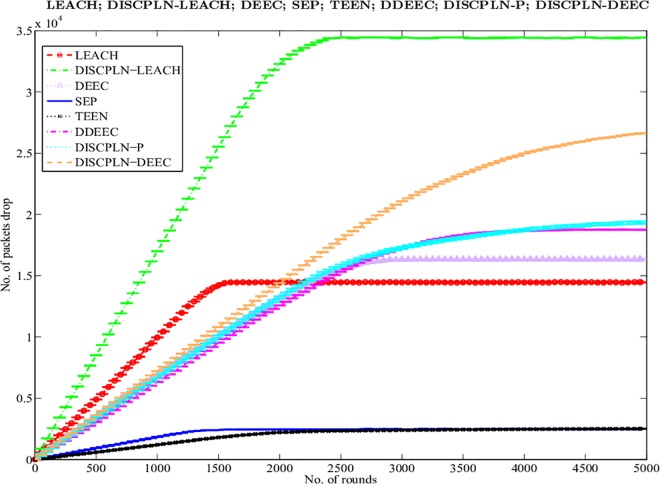
Number of packet drops for single-hop communication.

Ideally, when data packets move to the BS, they must all be received by the BS without any loss (i.e., total packets sent = total packets received). Packet drop occurs when some of these packets do not reach the BS. We simulated the results shown in Fig 8 using the random uniformed model to detect packet drops [57]. Following our assumption, a packet is dropped when the link status is below the required level for a successful reception. DISCPLN–LEACH exhibits the highest packet drop rate because of its probabilistic selection of CH, whereas LEACH shows the second highest packet drop rate when all of its nodes die. DISCPLN–P and DEEC have the same packet drop rates up to 2300 rounds. The packet drop rate of DISCPLN–DEEC is slightly higher than that of DEEC. Packet drop may also occur as a result of the variations in the residual energy of nodes for transmitting same-sized packets. Several data packets that are sent to a known destination may also be dropped because of the varying route length, the large packet size, and the nature of the route.

#### Delay time

**Fig 9:**
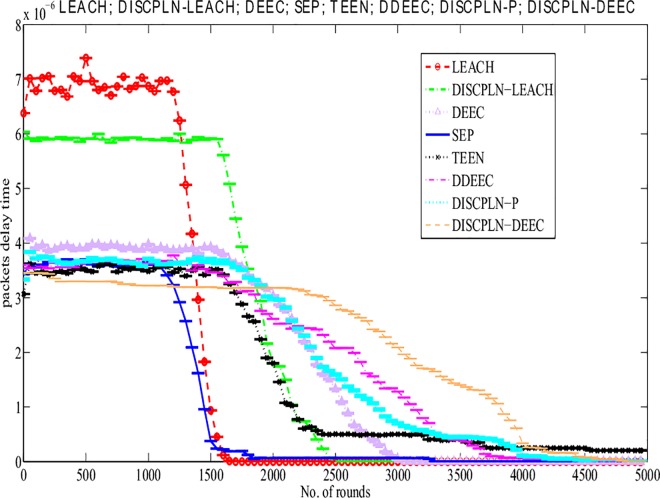
Number of delayed packets for single-hop communication.

LEACH has a longer delay time than DISCPLN–LEACH. As shown in Fig 9, delay time refers to the time that is spent in transmitting the packets from the sender to the receiver. DISCPLN–LEACH improves its performance based on network formation and node depletion in a specified region of a homogeneous network. DISCPLN–DEEC exhibits a short delay time because of the hierarchical distance-based communication in the heterogeneous network. Moreover, nodes in the internal region directly send their data to the BS with a short time delay, and this form of communication facilitates channel access.

## Proposed MH-DISCPLN: Multi-Hop Communication

**Fig 10:**
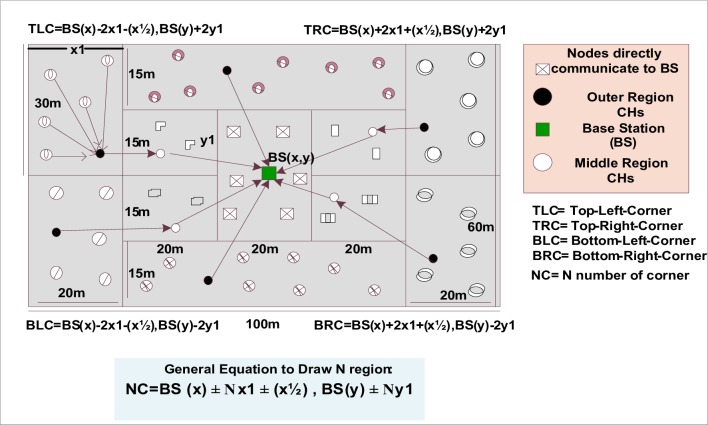
MH-DISCPLN model in homogeneous and heterogeneous environments.

Information in wireless networks is transferred from the source to the destination via two or more hops to achieve improved communication. In multi-hop communication, the nodes obtain the information at a considerably longer time. Instead of adopting a probabilistic selection of CHs, MH-DISCPLN introduces the novel concept of multi-hop clustering, in which an equal number of CHs are selected in each round. The simulation results of MH-DISCPLN are presented with extended area considerations, and the region formation and CH selection criteria are described in detail. MH-DISCPLN is combined with LEACH into MH-DISCPLN–LEACH in a homogeneous environment. By contrast, MH-DISCPLN is combined with DEEC into MH-DISCPLN–DEEC in a heterogeneous environment and then compared with DEEC, DDEEC, SEP, and TEEN. Each node contains the information of its fellow nodes. In this scenario, a certain number of nodes are considered and deployed over a regional dimension of M × M. We assume that the BS is placed (50, 30) at the center of the symmetric network area where the nodes are deployed in each region using the BS as a reference point. Fig 10 presents a detailed network model of MH-DISCPLN in homogeneous and heterogeneous environments.

### Region Formation

The network area is divided into internal, middle, and outer rectangles. The BS coordinates are considered reference points for marking a concentric rectangle (Fig 10). The BS is located in the internal rectangle, the outer region of the field with a size of 20 × 30 is called the outer rectangle, and the middle rectangle is placed at the center of both internal and outer rectangles with a size of 20 × 15. The concentric rectangle, except for the internal rectangle, is divided into different areas to specify the area of the network.

The four-corner coordinates of the network can be obtained as follows:

TLC of the network boundary (outer rectangle):

$$TLC=BS_{x}-2x_{1}-x/2,BS_{y}+y_{1},$$ (8)

TRC of the network boundary (outer rectangle):

$$TRC=BS_{x}+2x_{1}+x/2,BS_{y}+y_{1},$$ (9)

BLC of the network boundary (outer rectangle):

$$BLC=BS_{x}-2x_{1}-x/2,BS_{y}-y_{1},$$ (10)

BRC of the network boundary (outer rectangle):

$$BRC=BS_{x}+2x_{1}+x/2,BS_{y}-y_{1},$$ (11)

where *BS*_*x*,*y*_ is the location of the BS, *y*_1_ is the vertical distance from the BS to the outer rectangle of the network, and *x*_1_ is the horizontal distance from the BS to the internal rectangle boundary of the network. The factors *x*_1_ and *y*_1_ will be multiplied to draw more outer regions, and thus, enhance the network of the proposed scenario. *x*_1_ is multiplied by two to draw the outer rectangle of the network. The generalized equation (i.e., Eq 12) for the formation of the network field is expressed as follows:

$$NC=BS_{x}\pm Nx_{1}\pm x/2,BS_{y}\pm Ny_{1}.$$ (12)

### CH Selection

In the proposed technique, CHs are selected at two regions, namely, the middle and outer rectangles. The essential criteria for selecting the appropriate rectangular region are discussed in the subsequent subsections. The model is used in homogeneous and heterogeneous environments.

### Middle rectangle CH

- Clusters are dynamic, and clustering is static in every single round. Therefore, a single CH is selected in each cluster, except for the nodes of the internal rectangle.
- The nodes of the internal (rectangle) region directly transmit data to the BS.
- The node with the highest energy is selected as the CH.
- The node selected as CH cannot become a CH again until all the nodes in that region have become CH.
- The CHs of the middle rectangle aggregate and forward the data of their cluster members and the outer region CH to the BS.

### Outer rectangle CH

- The CH in the boundary region (outer rectangle) will transfer their received data to the nearest middle rectangle CHs.
- The selected CH cannot be a CH again until all the SNs in that region have become CH.

### Protocol Operation

Network establishment to data transmission operation is described in this section.

### Setup phase

- A uniform number of nodes is deployed into a network field, but these nodes are randomly distributed in each sector.
- Each SN knows its location.
- The entire network is distributed into small quadrilateral regions/rectangles based on the BS coordinates.
- The internal region (rectangle) directly sends its data to the BS.
- The CH is selected based on node residual energy.
- In a heterogeneous environment, advanced nodes become CHs more than normal nodes because their energy is *α* times higher than that of normal nodes.

### Steady-state phase

- Similar to LEACH and DEEC, each SN delivers its data to the CH within the assigned time slot via TDMA scheduling.
- The CH of the outer rectangle sends aggregated data to the CH of the middle rectangle.

### MH-DISCPLN Simulation Results

For consistency, the simulation parameters used are the same as those listed in Table 1, which are adopted in each scenario. The performance comparison of homogeneous protocols (e.g., LEACH and MH-DISCPLN–LEACH), as well as of heterogeneous protocols, is based on network lifetime and stability period.

#### Network lifetime

**Fig 11:**
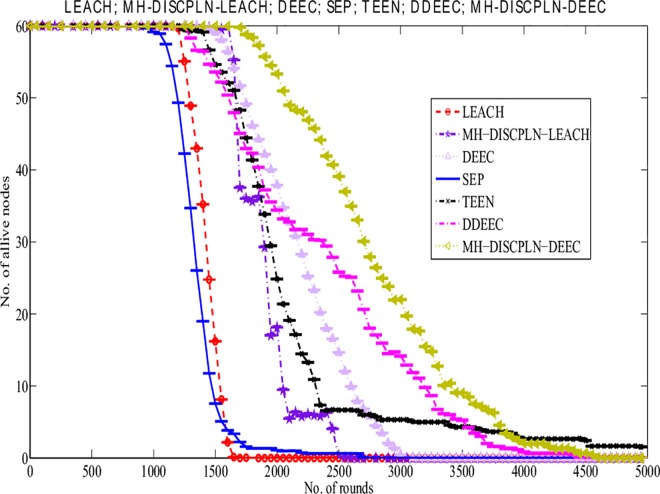
Performance comparison based on network stability period for multi-hop communication.

Fig 11 shows the evaluation of the lifetimes of the LEACH, DISCPLN–LEACH, DEEC, DDEEC, SEP, TEEN, and MH-DISCPLN–DEEC protocols. MH-DISCPLN–LEACH exhibited a higher stability period than the LEACH protocol. A clear difference of approximately 635 rounds is observed after area modification and node distribution in the specified region. In LEACH and MH-DISCPLN–LEACH, the first node died at round 944 and 1,565, respectively. The network lifetime of LEACH is generally less than that of MH-DISCPLN–LEACH. Energy is utilized efficiently and network stability period is prolonged in each region, except for the internal rectangle, by rotating the CHs during the formation process.

Among heterogeneous protocols, MH-DISCPLN–DEEC exhibits the maximum stability period because it considers the initial and residual energy of a node before selecting it as a CH. Meanwhile, TEEN has the largest unstable region. MH-DISCPLN–LEACH performs well compared with DDEEC, SEP, and TEEN. The node dies at round 4594 in MH-DISCPLN–DEEC, whereas the unstable region is observed at round 1429 in DEEC. In DEEC and SEP, varying the CHs in each round inefficiently aggregates data from all the nodes because randomly deploying nodes cannot cover the entire area. In our proposed protocol, a stable region is achieved because of the balanced selection of CHs. Moreover, the static clustering technique and multi-hopping conserve network energy and enhance network lifetime.

#### Throughput of the protocols

**Fig 12:**
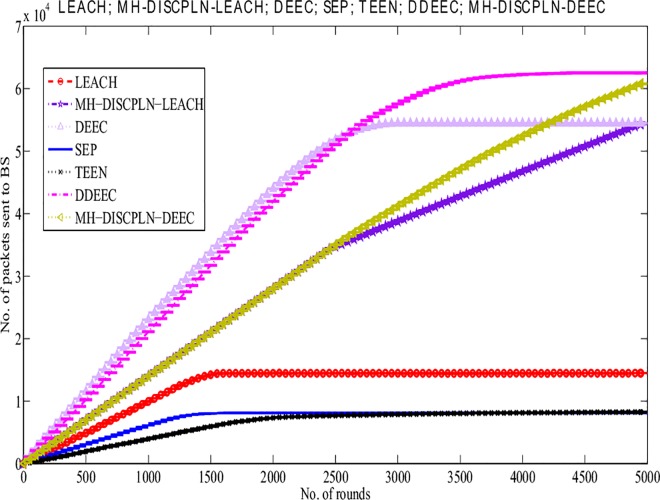
Performance comparison based on throughput for multi-hop communication.

A comparative study of the protocols is conducted to evaluate packet reception at the BS, and the results are presented in Fig 12. The increased packet reception rate is the result of the continuous data transmission of the internal rectangle nodes without CH formation. The comparison of LEACH and MH-DISCPLN–LEACH shows that the throughput of the latter increases linearly because CHs only transmit the associated SN data to the BS based on network division. However, after 2,425 rounds, throughput decreases slightly because live node density is significantly reduced during the final rounds. Thus, the dominant throughput of MH-DISCPLN–LEACH is authenticated based on Fig 12.

In case of a heterogeneous network, the throughput of MH-DISCPLN–DEEC lags behind those of DEEC and DDEEC. In the DEED and DDEEC protocols, a CH directly sends its data to the BS. By contrast, MH-DISCPLN–LEACH and MH-DISCPLN–DEEC send the information to the destination after aggregating the outer CH data to save the energy of the outer region nodes that are located far from the BS. However, in MH-DISCPLN–LEACH and MH-DISCPLN–DEEC, a linear and continuously increasing trend is observed because the same number of CHs is formed during each round. Moreover, LEACH, TEEN, and SEP present minimum throughput because of their network formation and communication nature.

#### Packet drop

**Fig 13:**
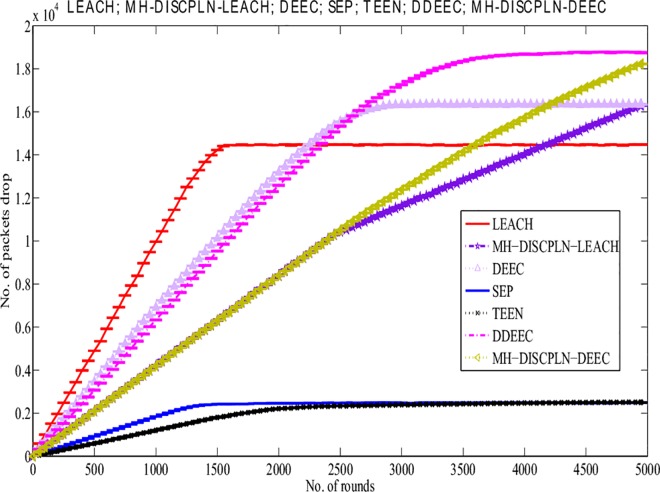
Performance comparison based on packet drop for multi-hop communication.

LEACH has a higher packet drop rate than MH-DISCPLN–LEACH because of its threshold-based CH selection. Moreover, MH-DISCPLN–LEACH and MH-DISCPLN–DEEC present the same results up to 2,500 rounds, although MH-DISCPLN–DEEC has a higher packet drop rate after this period. As shown in Fig 13, DEEC and DDEEC have higher packet drop rates than all the other protocols. Moreover, LEACH and DEEC with different cluster sizes in each round have higher packets drop rates than the proposed protocols.

#### Delay time

**Fig 14:**
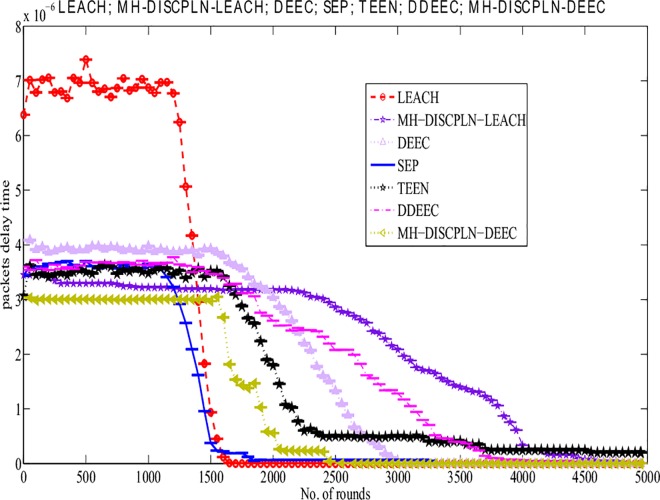
Performance comparison based on packet delay for multi-hop communication.

LEACH has the maximum delay time because of its clustering scheme. MH-DISCPLN–DEEC exhibits the shortest delay time (Fig 14) when hop-by-hop transmission is considered. Moreover, MH-DISCPLN–LEACH achieves the second best value in terms of minimum delay time. From the observation, we perceive that network deployment and clustering schemes directly affect packet delay time.

## Proposed M-DISCPLN: Sink Mobility-Based Protocol

A novel scheme based on sink mobility is introduced as M-DISCPLN. Network area divisions are similar to that of DISCPLN (Fig 4). However, data transmission is entirely different. In the proposed technique, the sink moves on a fixed path at a constant speed and stops at various positions to collect data from the nearest nodes. The mobile sink senses the data from all the sensors (because the CH also moves when it moves) and manages balanced energy consumption among nodes. With regard to sink mobility, several constraints are considered to prolong network lifetime. Blockage constraints are imposed on a maximum number of sink sojourn locations at a distance between two sojourn locations and the stop time for each location. Data loss has a high probability when the sink travels from one location to another location. Therefore, the distance between two sink locations should be bound. Nodes will only transmit data to the sink when it rests at the defined stop positions. If more than one sink location exists in the communication range of the nodes, then transmission will occur when a sink reaches the most feasible location. This criterion can enhance network lifetime. A comparative study of M-DISCPLN is conducted to evaluate the various performance parameters of the proposed network model. Furthermore, M-DISCPLN is simulated in homogeneous and heterogeneous environments through M-DISCPLN–LEACH and M-DISCPLN–DEEC, respectively. The system model, energy model, optimization problem, and lifetime maximization model for M-DISCPLN–LEACH are explained in the following subsections. Meanwhile, M-DISCPLN–DEEC nodes with different initial energy values, such as those in DEEC, are deployed.

### System Model for M-DISCPLN–LEACH

The problem is formulated based on mixed-integer linear programming. In the proposed model, we assume that the sink moves from Φ to location l. The network is modeled as a directed graph G = ϒ ∪ ϒ_0_, *ξ* ∪ *ξ*_0_, where | ϒ | = ℵ and ϒ_0_ is defined as the sink location set. ℵ represents the network nodes. *ξ* = ϒ ∪ ϒ is described as the set of links between SNs, and *ξ*_0_ is the set of links between nodes and sink locations. The term *ξ*_0_ = ϒ ∪ ϒ_0_ shows the wireless links between nodes and sink locations. We assume that the data generation rate is *θ*_*i*_ and the data rate is the same for all SNs. The description of all notations used is provided in Table 2.

**Table 2:** Notations and their description.

Notation	Description
ℵ	Set of static nodes
ϒ_0_	Set of sink stop locations
*η*_*n*_	Nodes nearest to the sink trajectory
*η*_*f*_	Nodes farthest from the sink trajectory
*θ*_*i*_	Data rate generated by node i
$z^{l}$	Sink sojourn time where l ∈ ϒ_0_
*E*_*i*_	Energy of node i
*ε*_*ij*_	Data transmission rate from node i to j
*ε*_*ki*_	Data reception rate while receiving from node i to node k
$D_{il}$	Minimum distance from node i to sink at location l
$\gamma_{l}$	Nodes transmitting data to sink at location l
*τ*	Time required for one epoch is *τ* s
*λ*_*i*_	Duration required to transmit data from node i to the mobile sink
ℑ_*ij*_	Upper bound on the transmission rate between link (i, j) ∈ ϒ_0_

The link between nodes and sink locations is $p_{il}$ when *i* and l are within the coverage range of the term $p_{il}$ = 1; otherwise, $p_{il}$ = 0, where ∀*i* ∈ ℵ. The total travel distance of the sink must be bound because it is mechanically driven by electricity or petrol. We also assume that, for recharging purposes, the sink starts and returns to a specific location denoted as Φ, which is located outside the network boundary.

The travel time between two sink sojourn locations is negligible, and the stability period of the sink at location l is $z^{l}$. Each node i generates the constant information *θ*_*i*_ until its energy E_*i*_ > 0. The aforementioned problem aims to enhance the network lifetime denoted by £, which is realized after collecting all the aggregated data from the sink sojourn locations.

Movement is preset in this proposed scheme sink. The mobile sink will obtain data by moving on its predetermined trajectory. At each location l, the nodes will only send data to the sink when the distance between the node and the sink is at its minimum. We categorize the nodes as the nearest nodes *η*_*n*_ and the farthest nodes *η*_*f*_ based on the distance from the mobile sink. This node notation will be helpful to minimize energy consumption.

### Energy Model

M-DISCPLN–LEACH is proposed in a homogeneous environment. All nodes have the same initial energy as calculated in Eq 13.

$$E_{i}=E_{0}$$ (13)

$$E_{ij}^{T}=\beta_{ij}^{t}.\varepsilon_{ij}$$ (14)

$$\beta_{ij}^{t}=\alpha +\varphi .d{\left(i,j\right)}^{e}$$ (15)

$$E_{ki}^{R}=\zeta .\varepsilon_{ki}$$ (16)

$$E_{total}=\sum_{j\in ℵ}E_{ij}^{T}+\sum_{k\in ℵ}E_{ki}^{R}=\sum_{j\in ℵ}\beta_{ij}^{t}.\varepsilon_{ij}+\sum_{k\in ℵ}\zeta .\varepsilon_{ki}$$ (17)

In constraint Eq 14, *E*^*T*^ is explained as the transmission energy of node i per epoch. $\beta_{ij}^{t}$ is the energy required to transmit data with rate *ε*_*ij*_ from node i to node j. $\beta_{ij}^{t}$ is calculated in Eq 15, where *α* and *φ* are the positive values, d is the distance between link i and j, and e is a path loss exponent based on the network environment. The constraint in Eq 16 shows that *ζ* is a constant value and *ε*_*ki*_ is the amount of energy consumed while receiving data from node i to node k. The constraint in Eq 17 denotes the total energy consumed while transmitting and receiving information.

### Optimization Problem

Network lifetime is maximized if the node has a minimum distance to the mobile sink because low energy will be required for data transmission. In M-DISCPLN–LEACH, the minimum distance is calculated using the optimization problem that derives feasible solutions as follows:

$$D_{il}=min[d(i,l).\lambda_{i}],$$ (18)

$$\chi_{i}=[\theta_{\tau }\lambda_{i},D_{il}(\gamma_{i}+1)\tau ],\;\forall l\in {ϒ}_{0},\;\forall i\in ℵ.$$ (19)

The constraint in Eq 18 presents the minimum distance from node i to the sink location at l at time duration *λ*_*i*_, in which node i transmits data with a data rate *θ*_*i*_ per epoch. In Eq 19, this data amount per epoch is denoted as *χ*_*i*_ and calculated as follows:

$${ℜ}_{total}=\sum_{i=1}^{ℵ}\chi_{i}.$$ (20)

In constraint Eq 20, ℜ_*total*_ is the amount of data collected by all nodes ℵ. This data amount is based on the number of nodes when the sink is at location l, which is also known as node density. If node density is lower at a specified location, then the mobile sink can easily gather data from all the nodes, as follows:

$$\gamma_{il}^{m}=\lceil \frac{\theta_{\tau }\lambda_{i}}{D_{il}}\rceil -1,\;\forall l\in {ϒ}_{0},\;\forall i\in ℵ,$$ (21)

$$\eta_{f}\geq \sum_{i=1}^{\eta_{n}}\gamma_{il}^{m},$$ (22)

$$\eta_{f}<\sum_{i=1}^{\eta_{n}}\gamma_{il}^{m},$$ (23)

$$\gamma_{i}\geq \gamma_{il}^{m},\forall i\in ℵ,$$ (24)

$$\gamma_{i}<\gamma_{il}^{m},\;\forall i\in ℵ,$$ (25)

where $\gamma_{il}^{m}$ is a requirement for the number of nodes for sink location l with the minimum distance from node i to sink location l, as shown in Eq 21. The constraint in Eq 22 shows that network density will be high when the number of member nodes is larger than that in Eq 23. The constraint in Eq 24 is the lower bound on nodes that satisfies the constraints in Eq 22. The sink increases network lifetime because of the reduced saturation of the nodes near the sink by changing its locations. For the low-density network in Eq 25, we assume that the sink at location l will not have more members than the network in Eq 24. Otherwise, the saturation of nodes will hinder the delivery of aggregated data to the mobile sink at a certain communication time. Thus, the problem can be formulated as follows:

Objective function:

$$min\sum_{i=1}^{ℵ}E_{i}$$ (26)

subject to:

$$\gamma_{s}\geq \gamma_{i}^{m},\;\forall s\in \eta_{n}\;if\;\eta_{f}\geq \sum_{i=1}\gamma_{il}^{m}$$ (27)

or

$$\gamma_{s}<\gamma_{i}^{m},\;\forall s\in \eta_{n}\;if\;\eta_{f}\geq \sum_{i=1}\gamma_{il}^{m},$$ (28)

$$\sum_{k=1}^{\eta_{n}}\gamma_{k}=\eta_{f},$$ (29)

$$D_{il}\leq d(i,l).\lambda_{i},\;\forall i\in ℵ,\;\forall l\in {ϒ}_{0}.$$ (30)

The problem focuses on reducing the overall energy consumption of the network, as shown in Eq 26. The constraints in Eqs 27 and 28 are bounds on the density of the network with a larger or smaller number of member nodes, which ensure that the sensed data will be obtained by the mobile sink. Meanwhile, the upper bound limit is satisfied by the relationship described in Eq 29. The constraint in Eq 30 limits the minimum distance between nodes and the mobile sink.

### Lifetime Maximization Model

The objective function of a mobile sink is to enhance the network lifetime as indicated in Eq 31. This objective is achieved by summing up all the locations of a sink, where the sink is stopped and the obtained data of node i lie within the transmission range in a specific region.

$$Maximize\;£=\sum_{l\in {ϒ}_{0}}z_{l}$$ (31)

subject to

$$\sum_{j\in ℵ(i,l)}\varepsilon_{ij}^{l}-\sum_{k:i\in ℵ(k,l)}\varepsilon_{ki}^{l}=\theta_{i},\;\forall i\in ℵ,\;\forall l\in {ϒ}_{0},$$ (32)

$$\sum_{l=1}^{{ϒ}_{0}}z_{l}(\sum_{j\in ℵ(i,l)}\beta_{ij}^{t}\varepsilon_{ij}^{l}-\sum_{k:i\in ℵ(k,l)}\zeta \varepsilon_{ki}^{l})\leq E_{i},\;\forall i\in ℵ,$$ (33)

$$\varepsilon_{ij}^{l}-{ℑ}_{ij}.z_{l}\leq 0,\;\forall (i,j),\;\forall l\in {ϒ}_{0},$$ (34)

$$\varepsilon_{ij}^{l}\geq 0,\;\forall i\in ℵ,\;\forall l\in {ϒ}_{0},\;\forall j\in ℵ(i,l),$$ (35)

$$z_{l}\geq 0,\;\forall l\in {ϒ}_{0}.$$ (36)

The constraint in Eq 32 shows that the outgoing data flow is equal to the incoming data flow of ℵ nodes for all the locations of the mobile sink. Incoming data flow is also based on the self-generated flow of a node, i.e., *θ*_*i*_. The constraint in Eq 33 presents the transmission energy and reception energy of ℵ sensors, which must not exceed the initial energy of a node. The total energy of the nodes is presented as the energy spent in the entire network lifetime. The constraint in Eq 34 shows that the total outgoing flow through links i and j should not exceed the capacity of link ℑ_*ij*_. The sink sojourn time required to transmit one unit of data from node i to node j should be greater than zero, as supported by Eqs 35 and 36.

### Simulation Results

A similar area is considered in a single-hop environment to compare the performance of M-DISCPLN–LEACH and M-DISCPLN–DEEC with those of LEACH, DISCPLN–LEACH, MH-DISCPLN–LEACH, DEEC, DICPLN–DEEC, DISCPLN–P, and MH-DISCPLN–DEEC. In this scenario, the WSN area is divided into five regions with randomly deployed nodes, and with each region containing an equal number of nodes. Initially, the sink location is considered outside the WSN for charging purposes. Then, the mobile sink travels at the predetermined trajectory for data aggregation, which enhances overall network lifetime.

**Fig 15:**
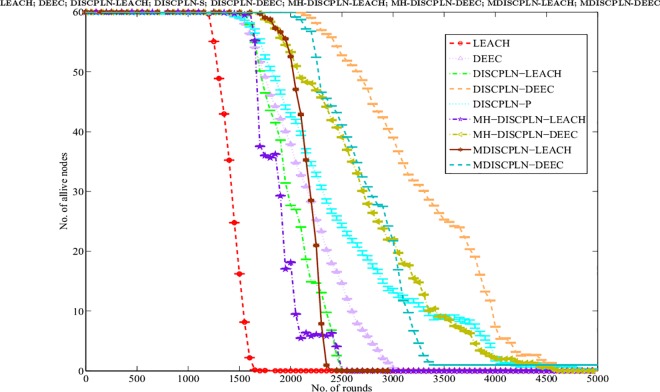
Performance comparison on the basis of alive nodes.

The M-DISCPLN–LEACH and M-DISCPLN–DEEC protocols are compared with the standard LEACH and DEEC protocols and the proposed protocols in the first two categories (Fig 15). M-DISCPLN has a mobile sink. In this protocol, the sink moves in each region and collects data from each node with the minimum distance to that node in every epoch. From the simulation results, DISCPLN–DEEC and M-DISCPLN–DEEC have stable regions up to 2,100 and 2,000 rounds, respectively. M-DISCPLN–LEACH is the third protocol that exhibits good performance. The unstable region of M-DISCPLN–LEACH starts at 1,700 rounds. When the mobile sink energy consumption of the nodes is minimized, network lifetime is enhanced because minimum distance reduces the number of transmissions and enhances the network stability period.

**Fig 16:**
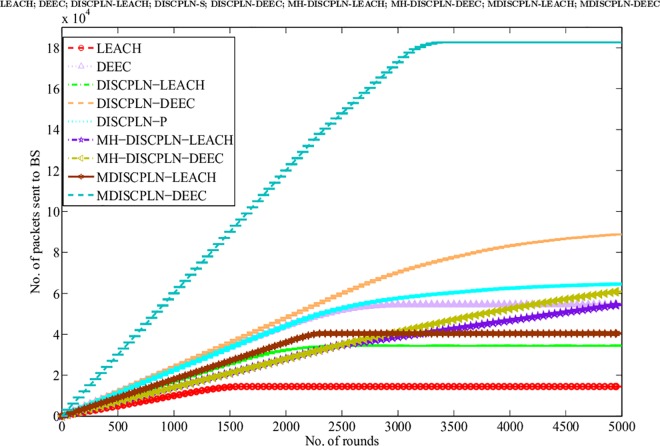
Performance comparison on the basis of network throughput.

The performance comparison based on network throughput (Fig 16) authenticates the prominent achievement in throughput. More packets are sent to the BS in M-DISCPLN–DEEC because every node verifies the minimum distance to the mobile sink. Node data are transmitted after finding a better location for the mobile sink.

## Comparison of the Overall Performances of Protocols

**Fig 17:**
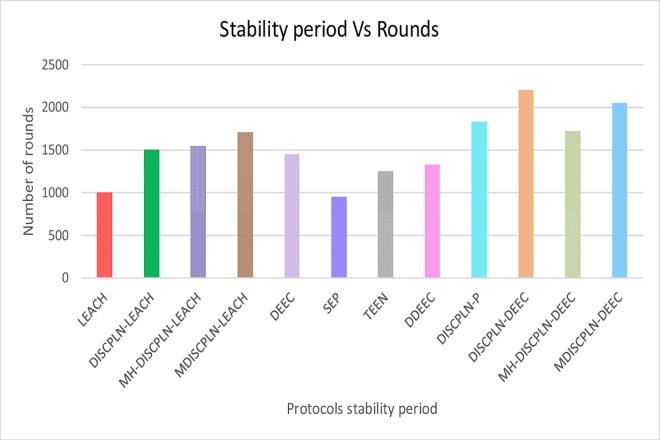
Performance comparison on the basis of network throughput.

This section presents the overall performance measures performed on and achieved by our proposed protocols in three different categories: single hop, multi-hop, and mobile sink. In the first category, a detailed network model is shown in Fig 4 for DISCPLN–LEACH and DISCPLN–DEEC. In that model, network formation, nodes, and depletion structure are elaborated. The overall simulation results are presented in Fig 17. Moreover, a comparative study of DISCPLN–LEACH and the conventional technique LEACH is provided in Fig 6 after executing the protocols in a same-sized network with an equal number of nodes. The performance of DISCPLN–LEACH is enhanced compared with that of LEACH. In the homogeneous environment, nodes are identical in nature because of battery power and physical structure. The simulation results show that, considering the efficient node deployment and CH formation strategy in each region except for the internal region, DISCPLN–LEACH improves the network lifetime. This observation is authenticated by the first node that died at 1,589 and 1,175 rounds for DISCPLN and LEACH, respectively. By contrast, the nodes in the heterogeneous environment are different because of their initial energy. DISCPLN-P and DISCPLN–DEEC with heterogeneous characteristics are compared with DEEC, DDEEC, SEP, and TEEN in the single-hop network environment. The enhanced CH selection criterion in DISCPLN–DEEC improves the performance of the technique. The comparative study shown in Fig 6 supports the enhanced stability characteristics of DISCPLN–DEEC, which has a stable region up to 2,203 rounds and exhibits improved response compared with DISCPLN-P at 1,830 rounds. In addition, DISCPLN-P is the second protocol that performs well compared with the other protocols.

A comparative study of the evaluation of network throughput, total number of packet drops during data transmission to the BS, and packet delay time is shown in Figs 7 to 9. The delay time of DISCPLN–LEACH is reduced compared with that of LEACH because of network deployment in regions. However, in heterogeneous networks, DISCPLN-P provides increased throughput based on the probabilistic approach similar to that in DEEC (Fig 7). Packet drop rate is reduced in each round, as shown in Fig 8. By contrast, DISCPLN–DEEC exhibits the minimum delay time because it guarantees an equal number of CH in each round that transmits to the BS. In the second category, our proposed technique MH-DISCPLN is presented for multi-hop transmission via a chain of CHs. The comparison of MH-DISCPLN with its counterparts supports the improved network lifetime and enhanced stability. MH-DISCPLN is further explained in homogeneous and heterogeneous networks via MH-DISCPLN–LEACH and MH-DISCPLN–DEEC, respectively, with the same network model for MH-DISCPLN. Fig 10 shows that the lifetime of MH-DISCPLN–LEACH and LEACH is 2,360 rounds and 1,650 rounds, respectively. The enhanced stable region of MH-DISCPLN–LEACH is 710 times that of LEACH in multi-hop communication. In the case of heterogeneous networks, the comparison with DEEC, DDEEC, SEP, and TEEN proves that the performance of MH-DICPLN–DEEC is slightly enhanced because an unstable region starts from 1,705 rounds (Fig 11). The throughput of MH-DISCPLN–LEACH is enhanced in the case of homogeneous networks. However, in heterogeneous environments, the throughput of DEEC and DDEEC is enhanced, as shown in Fig 12. Meanwhile, MH-DISCPLN–DEEC exhibits a shorter delay time compared with MH-DISCPLN–LEACH. The novel approach called M-DISCPLN with a mobile sink is presented as M-DISCPLN–LEACH and M-DISCPLN–DEEC, which are recommended for real-time systems.

The comparison of the performance of M-DISCPLN in homogeneous and heterogeneous networks with those of LEACH, DEEC, and our proposed protocols in the first two categories is illustrated in Fig 15. The comparison proves the stable characteristics of the proposed technique. Fig 16 validates that the throughput of M-DISCPLN–DEEC is higher because of sink mobility with minimum delay time. DISCPLN–DEEC performs well in terms of overall network lifetime. Moreover, M-DISCPLN is the second best protocol with the sink mobility concept. DISCPLN–DEEC and M-DISCPLN–DEEC slow down the death rate of their nodes while maintaining a long stability period compared with the well-known protocols, which have increasing death rates. In addition, too few and too many CHs affect network lifetime. Consequently, the CHs of LEACH, DEEC, and TEEN are selected based on probability and CH. By contrast, a uniform cluster size ensures good network performance as shown in our proposed protocols. Finally, we conclude from the results of the comparison that an equal number of nodes and a similar network arena improve performance with different proposed network structures and topologies. The enhanced network lifetime providesinformation for a long period.

## Conclusion

Energy-aware clustering algorithms enhance network performance in WSNs. Inevitably, an unequal cluster size significantly affects energy consumption because of the non-uniform distribution of nodes. Therefore, we propose the DISCPLN and MH-DISCPLN protocols in this work to enhance network performance. DISCPLN and MH-DISCPLN are energy-aware physical clustering and distance-based protocols used in homogenous and heterogeneous networks. DISCPLN is simulated with single-hop communication, whereas MH-DISCPLN is simulated with multi-hop communication. We investigate the issue of unequal selection of a CH in uniformly distributed sensor networks based on distance. Extensive simulations show that DISCPLN and MH-DISCPLN extend network lifetime by 30% compared with existing benchmark protocols. Thus, nodes should be equally distributed and clusters in network areas should be controlled to avoid the coverage hole problem. Moreover, the behavior of M-DISCPLN protocols with a mobile sink without CH formation is observed. In M-DISCPLN, the mobile sink balances the energy load among nodes and obtains more data from nodes to increase network lifetime. This study focuses on different protocols proposed to overcome the coverage hole and efficient energy utilization issues while maximizing network lifetime by evenly distributing the load across the entire network nodes in different regions. Thus, improving the stability period through efficient energy utilization remains an open issue. Moreover, the detection and prevention of energy holes are interesting research problems if the nodes are not anchored. However, we will consider a real-time experimental test bed development for mobile and static sensor networks in the future.

## Supporting Information

S1 Fig S1 Fig shows the node deployment in the conventional protocol. (EPS)

S2 Fig S2 Fig presents the WSN tunnel-based application. (EPS)

S3 Fig S3 Fig shows the radio energy dissipation model. (EPS)

S4 Fig S4 Fig shows the DISCPLN protocol model in homogeneous and heterogeneous environments. (EPS)

S5 Fig S5 Fig shows the DISCPLN–DEEC CH selection. (EPS)

S6 Fig S6 Fig shows the Performance comparison based on stability period for single-hop communication. (EPS)

S7 Fig S7 Fig shows the performance comparison based on throughput for single-hop communication. (EPS)

S8 Fig S8 Fig shows the number of packet drops for single-hop communication. (EPS)

S9 Fig S9 Fig shows the Number of delayed packets for single-hop communication S9 Fig. (EPS)

S10 Fig S10 Fig shows the MH-DISCPLN model in homogeneous and heterogeneous environments. (EPS)

S11 Fig S11 Fig shows the performance comparison based on network stability period for multi-hop communication. (EPS)

S12 Fig S12 Fig shows the performance comparison based on throughput for multi-hop communication. (EPS)

S13 Fig S13 Fig shows the performance comparison based on packet drop for multi-hop communication. (EPS)

S14 Fig S14 Fig shows the performance comparison based on packet delay for multi-hop communication. (EPS)

S15 Fig S15 Fig shows the performance comparison on the basis of alive nodes. (EPS)

S16 Fig S16 Fig shows the performance comparison on the basis of network throughput. (EPS)

S17 Fig S17 Fig shows the performance comparison on the basis of network throughput. (EPS)

S1 Table This is the S1 Table. (DOCX)

S2 Table This is the S2 Table. (DOCX)
