# Supplementary material for: Distance-Based and Low Energy Adaptive Clustering Protocol for Wireless Sensor Networks
Source: PLoS One. 2016 Sep 22;11(9):e0161340. doi: 10.1371/journal.pone.0161340 (PMC5033373; doi:10.1371/journal.pone.0161340)
Supplement: S1 Table — (DOCX) [file pone.0161340.s018.docx]

| **Parameters** | **Values** |
| --- | --- |
| Network Size | 100 m × 60 m |
| Sensor Nodes | 60 |
| BS | (50 m, 30 m) |
| Packet Size | 4000 bits |
| Initial Energy $(E_{0})$ | 0.5 J |
| Dissipated Energy per Bit $(E_{elec})$ | 50 nJ/bit |
| Probability of Packet Drop | 0.3 |

Table 1: Simulation parameters
