# Supplementary material for: Distance-Based and Low Energy Adaptive Clustering Protocol for Wireless Sensor Networks
Source: PLoS One. 2016 Sep 22;11(9):e0161340. doi: 10.1371/journal.pone.0161340 (PMC5033373; doi:10.1371/journal.pone.0161340)
Supplement: S2 Table — (DOCX) [file pone.0161340.s019.docx]

**Table 2: Notations and their description**

| **Notation** | **Description** |
| --- | --- |
| $\aleph$ | Set of static nodes |
| $ϒ_{0}$ | Set of sink stop locations |
| $\eta_{n}$ | Nodes nearest to the sink trajectory |
| $\eta_{f}$ | Nodes farthest from the sink trajectory |
| $\theta_{i}$ | Data rate generated by node i |
|  | Sink sojourn time where $\in$ $ϒ_{0}$   |
| $E_{i}$ | Energy of node $\text{i}$ |
| $\varepsilon_{ij}$ | Data transmission rate from node $\text{i}$ to $\text{j}$ |
| $\varepsilon_{ki}$ | Data reception rate while receiving from node $\text{i}$ to node $\text{k}$ |
|  | Minimum distance from node $\text{i}$ to sink at location  |
|  | Nodes transmitting data to sink at location  |
| $\tau$ | Time required for one epoch is $\tau$ s |
| $\lambda_{i}$ | Duration required to transmit data from node i to the mobile sink |
| $\Im_{ij}$ | Upper bound on the transmission rate between link $\text{(i,j)}$ $\in$ $ϒ_{0}$   |
